# Dynamic Electromagnetic Scattering Simulation of Tilt-Rotor Aircraft in Multiple Modes

**DOI:** 10.3390/s23177606

**Published:** 2023-09-01

**Authors:** Zhongyang Fei, Yan Yang, Xiangwen Jiang, Qijun Zhao, Xi Chen

**Affiliations:** National Key Laboratory of Helicopter Aeromechanics, College of Aerospace Engineering, Nanjing University of Aeronautics and Astronautics, Nanjing 210016, China; feizhongyang@nuaa.edu.cn (Z.F.); yangyan1982@nuaa.edu.cn (Y.Y.); jiangxiangwen@nuaa.edu.cn (X.J.); chenxicc@nuaa.edu.cn (X.C.)

**Keywords:** tilt-rotor aircraft, multiple modes, dynamic electromagnetic scattering, radar cross section, micro-Doppler, time-varying mesh method

## Abstract

To study the electromagnetic scattering of tilt-rotor aircraft during multi-mode continuous flight, a dynamic simulation approach is presented. A time-varying mesh method is established to characterize the dynamic rotation and tilting of tilt-rotor aircraft. Shooting and bouncing rays and the uniform theory of diffraction are used to calculate the multi-mode radar cross-section (RCS). And the scattering mechanisms of tilt-rotor aircraft are investigated by extracting the micro-Doppler and inverse synthetic aperture radar images. The results show that the dynamic RCS of tilt-rotor aircraft in helicopter and airplane mode exhibits obvious periodicity, and the transition mode leads to a strong specular reflection on the rotor’s upper surface, which increases the RCS with a maximum increase of about 36 dB. The maximum micro-Doppler shift has functional relationships with flight time, tilt speed, and wave incident direction. By analyzing the change patterns of maximum shift, the real-time flight state and mode can be identified. There are some significant scattering sources on the body of tilt-rotor aircraft that are distributed in a planar or point-like manner, and the importance of different scattering sources varies in different flight modes. The pre-studies on the key scattering areas can provide effective help for the stealth design of the target.

## 1. Introduction

Due to the advantages of vertical take-off and landing, high speed, and long range, tilt-rotor aircraft are playing an increasingly important role in many fields, and with that come higher requirements for their radar stealth. With the conversion of flight modes, tilt-rotor aircraft exhibit complex and variable scattering features, which makes the design work of radar stealth more difficult. So, it is of great significance to investigate the dynamic electromagnetic scattering characteristics of the tilt-rotor aircraft in multiple modes.

Among the various electromagnetic scattering characteristics of a vehicle, the most important is the radar cross-section (RCS), whose reduction is crucial to the survivability of the vehicle [1,2]. But the tilt-rotor aircraft has two rotors that can both rotate and tilt, which can have special impacts on the electromagnetic wave modulation [3]. Therefore, it is also important to study the micro-Doppler effects of tilt-rotor aircraft.

In the RCS calculation for an electrically large and structurally complex vehicle, the shooting and bouncing rays (SBR) method is often used to track the multiple reflection paths of the radar rays and solve the surface reflection [4], and the uniform theory of diffraction (UTD) is used to calculate the edge diffraction [5]. By mixing the different high-frequency prediction methods, calculation accuracy and efficiency can be effectively ensured [6,7,8]. Unlike fixed-wing aircraft, rotorcraft have rotating parts such as rotors and tail rotors that are always in a high-speed rotating state. For this reason, Bladel first proposed the quasi-static principle (QSP) for the scattering analyses of the rotating parts of rotorcraft [9], which provides a basis for studying the dynamic RCS of rotorcraft. However, it is difficult to analyze the dynamic electromagnetic scattering characteristics of the tilt-rotor aircraft in multiple modes by only relying on the basic QSP because the aircraft can simultaneously tilt the nacelles at low speed and rotate the two rotors at high speed with opposite rotation directions [10]. Therefore, it is necessary to establish a method of dynamic time-varying mesh that can accurately characterize the shape of the tilt-rotor aircraft at any moment, so as to clearly simulate the dynamic process [11,12].

In addition, the position relationship between the rotating and static parts of a conventional helicopter or a propeller plane is usually constant, and the rotating parts only rotate around one axis, so that the micro-Doppler frequency shift can be easily estimated by obtaining the angle between the rotation disk and the radar rays [13]. Due to the diversity of its flight modes, only in helicopter or airplane mode does the tilt-rotor aircraft have a simple micro-Doppler feature [14]. When the tilt-rotor aircraft flies in transition mode, its rotors and nacelles perform non-coaxial and coupled rotational motions [15,16]. These lead to the time-varying change of the position relationships between the rotors, nacelles, and other static parts and aggravate the difficulty of extracting the modulation characteristics and the complexity of micro-Doppler features in the transition mode. The tilting of rotors also changes the strong scattering sources and brings different RCS patterns. So, microwave imaging means are required to get the distribution of the strong scattering sources in multiple modes and to provide guidance for the stealth design of the tilt-rotor aircraft.

To date, some RCS analyses of tilt-rotor aircraft have been conducted under specific conditions, but few studies have focused on the dynamic electromagnetic scattering characteristics in the continuous process from helicopter mode to transition mode and then to airplane mode. In view of the above, in this paper, the RCS hybrid algorithm combining SBR and UTD is first established. The dynamic time-varying mesh approach is proposed to characterize the multi-mode motions of the tilt-rotor aircraft, and then the dynamic RCS responses are calculated. Next, the micro-Doppler and the scattering source distribution are extracted by short-time Fourier transformation (STFT) and inverse synthetic aperture radar (ISAR) imaging, and the effects of the attitude angles are investigated. Furthermore, the guidance significance of the dynamic scattering characteristics on the stealth design and the target identification of the tilt-rotor aircraft are discussed.

## 2. Dynamic Scattering Simulation Method of Tilt-Rotor Aircraft

### 2.1. Methods of RCS Calculation

The SBR method is based on the classical ray tube theory, which determines the incidence propagation path by ray tracing and then solves the integral of the scattering field. The SBR is often used to quickly calculate the RCS of large and complex targets (e.g., aircraft and ships). However, the SBR is unable to compute the edge diffraction of the target, so the UTD method is introduced for correction. The total scattering field of the target can be obtained by vector superposition of the surface reflection calculated by SBR and the edge diffraction calculated by UTD. The combination of the two methods significantly improves computational efficiency and accuracy.

**Figure 1:**
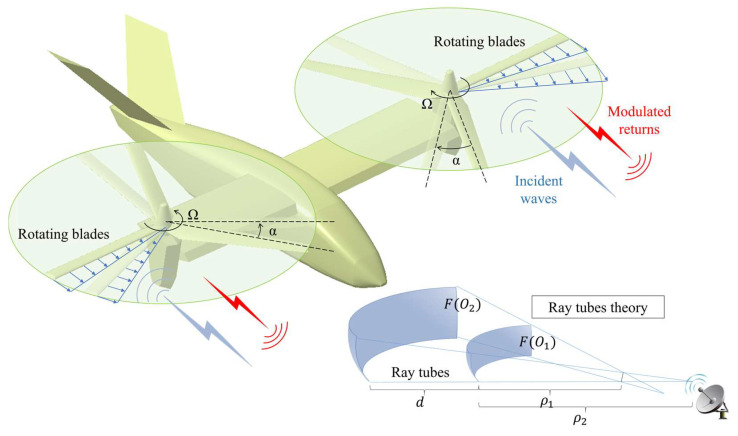
Diagram of dynamic electromagnetic scattering of tilt-rotor aircraft.

The SBR method first generates ray tubes, tracks the wave propagation paths according to the principle of GO, and then solves for the RCS based on the PO integral equations. Under the approximation conditions of GO, the electromagnetic wave is regarded as a local plane wave, and the energy propagates along the ray tubes, as shown in Figure 1. According to the energy conversation law and Snell’s reflection law, at any point on the surface element of the target, the reflection field $E_{r}$ satisfies:

(1) $$E_{r}(d_{n+1}^{\prime })=\sqrt{\frac{\rho_{1}\rho_{2}}{\left(\rho_{1}+d)(\rho_{2}+d\right)}}\cdot \Gamma \cdot E_{i}(d_{n}^{\prime })\cdot e^{-jk_{0}d^{\prime }},$$

where d is the distance from wave front $F\left(O_{1}\right)$ to $F\left(O_{2}\right)$, $\rho_{1}$ and $\rho_{2}$ are the two principal curvatures of wave front $F\left(O_{1}\right)$, $E_{i}$ is the incident field, Γ is the reflection coefficient of target surface, and $d^{\prime }$ denotes the propagation path of the reflected rays, $d^{\prime }=\left|d_{n+1}^{\prime }-d_{n}^{\prime }\right|$, $e^{-jk_{0}d^{\prime }}$ is the propagation phase factor, $k_{0}$ is the free-space wave number, and j is an imaginary unit.

Based on the Stratton–Chu integral equation, PO replaces the scatterers with the induced currents, and the scattering electric field at any point “P” outside the target is expressed as [17]:

(2) $$E_{s}(P)=\oint \left[j\omega \mu (n\times H_{t})\psi +(n\times E_{t})\times \nabla \psi +(n\cdot E_{t})\nabla \psi \right]ds,$$

where μ is the magnetic permeability, ω is the angle frequency of the electromagnetic wave, n is the unit normal vector at the scatterer’s surface, $H_{t}$ and $E_{t}$ are the total magnetic field and total electric field at the scatterer’s surface, respectively, ψ is the free space Green’s function, and ds is the surface micro-element.

Under the three approximation conditions of high frequency, far field, and tangent plane, the Stratton–Chu equation can be transformed into:

(3) $$E_{s}(P)=\frac{j\omega \mu e^{jk_{0}R_{0}}}{2\pi R_{0}}\oint \left[(n\times H_{i})-k_{0}^{s}\times (n\times H_{i})k_{0}^{s}\right]\cdot e^{-jk_{0}k_{0}^{s}r_{0}}ds_{i},$$

where $R_{0}$ is the distance from the scatterer to the radar antenna, $k_{0}^{s}$ is the unit vector in the direction of scattering, $ds_{i}$ is the surface micro-element in the illuminated area, $r_{0}$ is the distance from $s_{i}$ to the radar antenna, and $H_{i}$ is the incident magnetic field.

The SBR method can quickly calculate the surface reflection, but it fails when calculating the edge diffraction, so the method of equivalent currents based on the UTD form [18] is introduced for correction. This method assumes that there are equivalent currents and magnetic currents at the edges, and the edge diffraction can be obtained by the radiation integration of the equivalent currents and magnetic currents. The general form of the edge diffraction integral formula is given by [19]:

(4) $$E_{d}=-jk_{0}\psi \int \left[Z_{0}I_{e}s\times (s\times t)+I_{m}(s\times t)\right]\cdot e^{-jk_{0}r\cdot s}dl,$$

where dl is the micro-element on the edge, $Z_{0}$ is the wave impedance, s is the unit normal in the direction of the diffraction ray, r is the position vector of any point on the edge, t is the unit tangent vector on the edge, $I_{e}$ and $I_{m}$ are equivalent electric currents and magnetic currents, respectively.

Then, the total scattering fields of the target can be obtained by vector superposition of the surface reflection fields and the edge diffraction fields, and the RCS of the tilt-rotor aircraft can be further calculated.

### 2.2. Principle of Micro-Doppler and ISAR Imaging

The magnitude of the RCS only shows the echo intensity of the target in a certain state. The micro-Doppler spectrum and microwave imaging, on the other hand, are able to reveal the dynamic electromagnetic scattering phenomenon of the tilt-rotor aircraft in transition mode from multiple perspectives, including time, frequency, and space.

The rotating rotors will have a frequency modulation effect on the radar waves. The time-domain echo signal can be converted into an energy spectrum distribution in the time-frequency domain by STFT. The discrete form of the STFT is as follows:

(5) $$\mathrm{STFT}(mT,F_{0})=\sum_{k=-\infty }^{k=+\infty }z(k)g^{*}(kT-mT)\;e^{-jF_{0}k},$$

where T is the time sampling period, $F_{0}$ is an integer multiple of the frequency sampling period, $z\left(k\right)$ represents discrete signals, m and k are integers, $g^{*}$ is the time window function. In this paper, the Hamming window function is used.

The maximum frequency shift caused by the rotating rotors is:

(6) $$F_{\mathrm{dmax}}=\frac{2R\Omega \cos\theta_{0}}{\lambda },$$

where R is the radius of rotors, Ω is the rotation speed, $\theta_{0}$ is angle between incident direction and rotor disk, and λ is the wavelength.

And the time sampling period needs to satisfy the Nyquist sampling theorem:

(7) $$T\leq \frac{1}{2F_{\mathrm{dmax}}},$$

**Figure 2:**
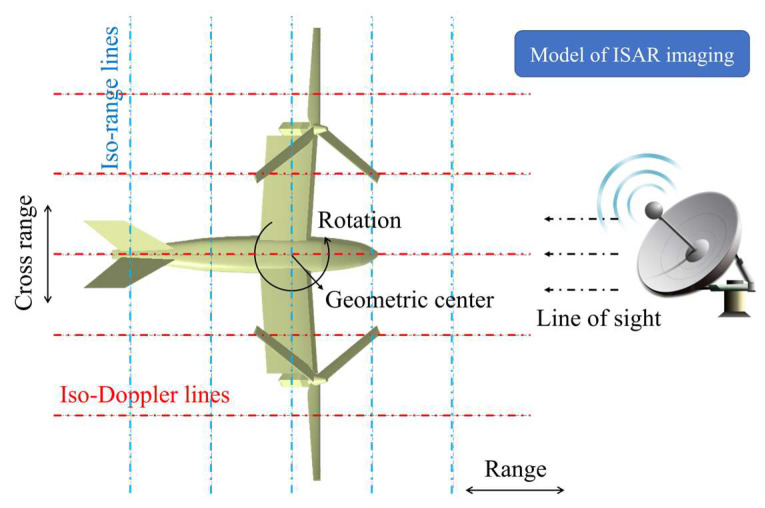
Model of ISAR imaging for tilt-rotor aircraft.

ISAR imaging based on the turntable model can project the electromagnetic scattering of the rotor on a two-dimensional coordinate system and clearly characterize the scattering intensity of different parts of the tilt-rotor aircraft. The two-dimensional coordinate system includes the range coordinate along the line of sight of radar and the cross-range coordinate perpendicular to the line of sight of radar, which should satisfy the following relationships for clear imaging:

(8) $$\left\{\begin{array}{c} \rho_{a}^{2}>\frac{\lambda D_{r}}{4}\\ \rho_{a}\rho_{r}>\frac{\lambda D_{a}}{4} \end{array}\right.,$$

where $\rho_{r}$ is the range resolution, $\rho_{a}$ is the cross-range resolution, $D_{r}$ is the size of the target’s range, $D_{a}$ is the size of the target’s cross-range. Figure 2 shows the model of ISAR imaging for tilt-rotor aircraft.

### 2.3. Dynamic Process Simulation

The research object of this paper is a twin-rotor, V-tail tilt-rotor aircraft that has pointed splits on the leading edges of wings, polyhedral nacelles, and tail wings, and has streamlined profiles on the rotors and fuselage. The main parameters of the tilt-rotor aircraft model are shown in Table 1. And the tilt-rotor aircraft is assumed to be made of PEC (perfect electric conductor).

#### 2.3.1. Principle of Quasi-Static

The tilt-rotor aircraft has two rotors with the same rotation speed and opposite rotation directions, and its model is always changing periodically. In order to accurately describe the dynamic motions of the tilt-rotor aircraft, the dynamic motion process is discretized into a series of quasi-static states:

(9) $$S=\left\{s(t_{1}),\;s(t_{2}),\;\ldots ,\;s(t_{N})\right\},$$

where S represents the dynamic motion process, s represents the quasi-static states, N is the number of quasi-static states, t is the discrete moments.

And the time interval between the two adjacent discrete moments is the same, expressed as:

(10) $$\Delta t=t_{n}-t_{n-1},\;n=2,3,\ldots ,N-1,N,$$

#### 2.3.2. Dynamic Coordinates Transformation

**Figure 3:**
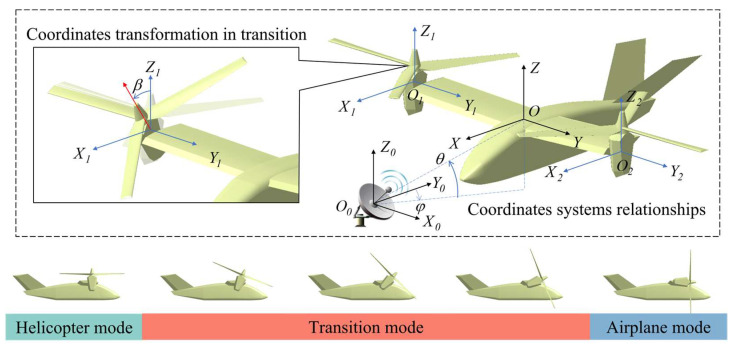
Coordinate systems transformation and flight modes transformation.

Converting the dynamic model of the tilt-rotor aircraft to mesh information is required for RCS calculation. The dynamic change of the meshes is based on the transformation of multiple coordinate systems. Figure 3 demonstrates the definitions of different Cartesian coordinate systems. Where $O_{0}X_{0}Y_{0}Z_{0}$ is the radar coordinate system, OXYZ is the aircraft body coordinate system whose ZX plane is located in the symmetrical plane. $O_{1}X_{1}Y_{1}Z_{1}$ is the right nacelle tilting coordinate system, $O_{2}X_{2}Y_{2}Z_{2}$ is the left nacelle tilting coordinate system, the $Y_{1}$ and $Y_{2}$ axes are the tilting axes, β is the tilting angle. The Y, $Y_{1}$ and $Y_{2}$ axes lie on the same line. And φ is the azimuth angle of the incident wave, θ is the pitch angle.

Let the grid coordinate matrix of the tilt-rotor aircraft under the aircraft body coordinate system at the initial moment be represented as:

(11) $$M\left(t_{0}\right)=\left\{M_{f}\left(t_{0}\right),\;M_{r,r}\left(t_{0}\right),\;M_{r,l}\left(t_{0}\right),\;M_{n,r}\left(t_{0}\right),\;M_{n,l}\left(t_{0}\right)\right\},$$

where $M_{f}$, $M_{r,r}$, $M_{r,l}$, $M_{n,r}$ and $M_{n,l}$ are the grid coordinate matrices of the fuselage, right rotor, left rotor, right nacelle, and left nacelle under the aircraft body coordinate system, respectively.

At different moments, we have a rotation angle of $\alpha \left(t\right)$ and a tilting angle of $\beta \left(t\right)$. Then

(12) $$M_{f}\left(t\right)=M_{f}\left(t_{0}\right),$$

(13) $$M_{r,r}\left(t\right)=A^{-1}\cdot \left[\begin{array}{ccc} \cos\beta \left(t\right) & 0 & \sin\beta \left(t\right)\\ 0 & 1 & 0\\ -\sin\beta \left(t\right) & 0 & \cos\beta \left(t\right) \end{array}\right]\left[\begin{array}{ccc} \cos\alpha \left(t\right) & -\sin\alpha \left(t\right) & 0\\ \sin\alpha \left(t\right) & \cos\alpha \left(t\right) & 0\\ 0 & 0 & 1 \end{array}\right]A\cdot M_{r,r}\left(t_{0}\right),$$

(14) $$M_{r,l}\left(t\right)=A\cdot \left[\begin{array}{ccc} \cos\beta \left(t\right) & 0 & \sin\beta \left(t\right)\\ 0 & 1 & 0\\ -\sin\beta \left(t\right) & 0 & \cos\beta \left(t\right) \end{array}\right]\left[\begin{array}{ccc} \cos\alpha \left(t\right) & \sin\alpha \left(t\right) & 0\\ -\sin\alpha \left(t\right) & \cos\alpha \left(t\right) & 0\\ 0 & 0 & 1 \end{array}\right]A^{-1}\cdot M_{r,l}\left(t_{0}\right),$$

(15) $$M_{n,r}\left(t\right)=A^{-1}\cdot \left[\begin{array}{ccc} \cos\beta \left(t\right) & 0 & \sin\beta \left(t\right)\\ 0 & 1 & 0\\ -\sin\beta \left(t\right) & 0 & \cos\beta \left(t\right) \end{array}\right]A\cdot M_{n,r}\left(t_{0}\right),$$

(16) $$M_{n,l}\left(t\right)=A\cdot \left[\begin{array}{ccc} \cos\beta \left(t\right) & 0 & \sin\beta \left(t\right)\\ 0 & 1 & 0\\ -\sin\beta \left(t\right) & 0 & \cos\beta \left(t\right) \end{array}\right]A^{-1}\cdot M_{n,l}\left(t_{0}\right),$$

where A is the transformation matrix from OXYZ to $O_{1}X_{1}Y_{1}Z_{1}$, $A^{-1}$ is the transformation matrix from OXYZ to $O_{2}X_{2}Y_{2}Z_{2}$. After that, the grid coordinate matrix of the whole tilt-rotor aircraft at different moments can be updated to

(17) $$M\left(t\right)=\left\{M_{f}\left(t\right),\;M_{r,r}\left(t\right),\;M_{r,l}\left(t\right),\;M_{n,r}\left(t\right),\;M_{n,l}\left(t\right)\right\},$$

**Figure 4:**
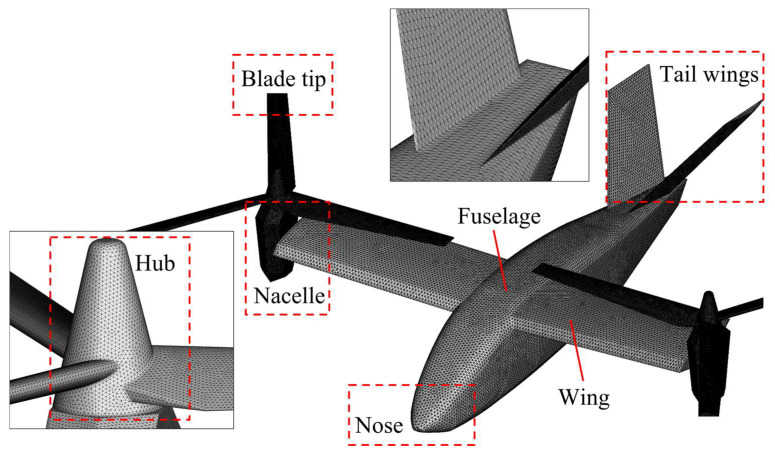
Surface meshes of the tilt-rotor aircraft.

The mesh information is constantly updated and stored with time, which can realize dynamic process simulation and real-time RCS calculations. When generating the dynamic meshes, the degree to which the meshes fit the aircraft model determines the calculation accuracy. So, the meshes are encrypted at the rotating parts and the places where the shape changes drastically, as shown in Figure 4.

For the above tilt-rotor aircraft model, surface meshes of about 244,000 are generated, and the RCS calculation is carried out based on the high-frequency prediction algorithm established in Section 2.1. It takes about 39 s to calculate a state point. The computer configuration is as follows: AMD Ryzen 9 7900X processor, 12 cores, 4.7 GHz CPU basic frequency on Windows x64 operating system.

### 2.4. Method Verification

**Figure 5:**
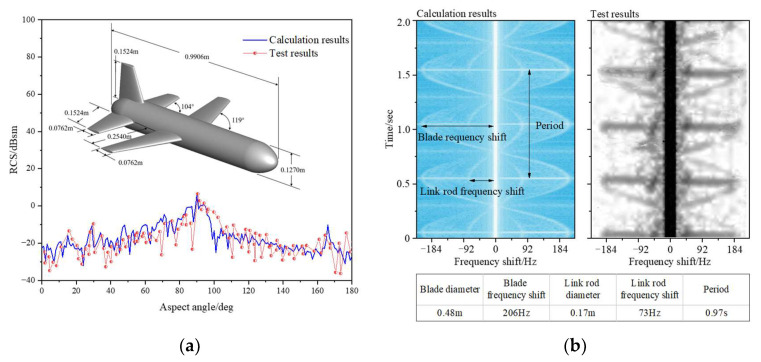
Validation of the dynamic scattering simulation method. (**a**) General missile RCS; (**b**) rotor micro-Doppler.

In this section, the dynamic simulation method is verified by a general missile with RCS test results [20] and a rotor with micro-Doppler test results [21]. The general missile has a pitch angle of −10.5° and a roll angle of 0°, and the radar works at 12 GHz and HH polarization. The rotor has two blades and rotates at a speed of 60 r/min, and the radar works at 10 GHz and HH polarization. From Figure 5a,b, it can be seen that the RCS calculation results of the general missile and the micro-Doppler calculation results of the rotor are both in good agreement with the test results, indicating that the simulation method can be effectively applied to the study of the dynamic electromagnetic scattering of the tilt-rotor aircraft.

## 3. Results and Analyses

### 3.1. Multi-Mode Dynamic Electromagnetic Scattering

**Figure 6:**
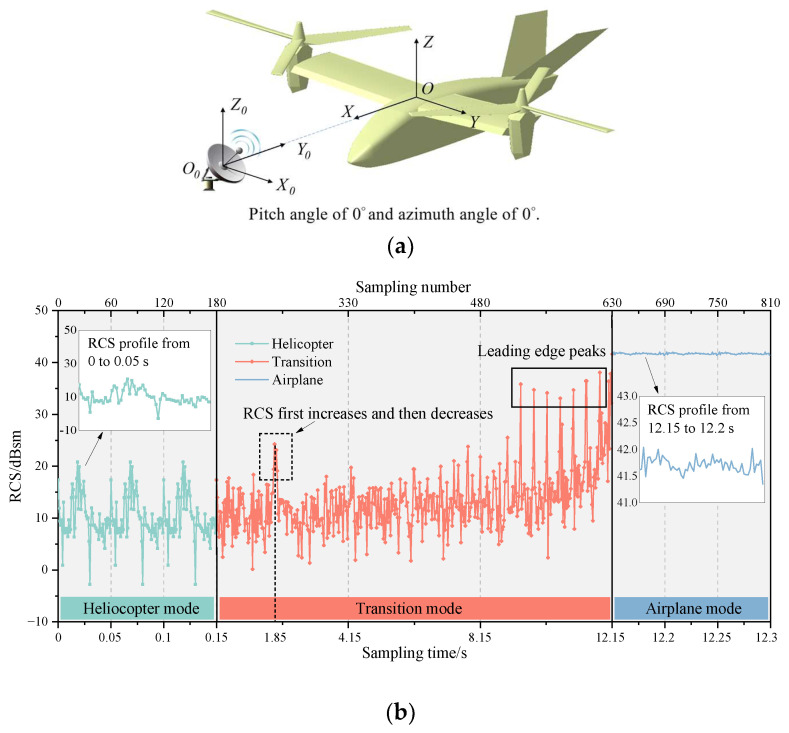
Multi-mode dynamic RCS of tilt-rotor aircraft, $\theta =\varphi =0^{\circ }$, f=10GHz. (**a**) Relative directional relationship between aircraft and radar; (**b**) dynamic RCS.

**Figure 7:**
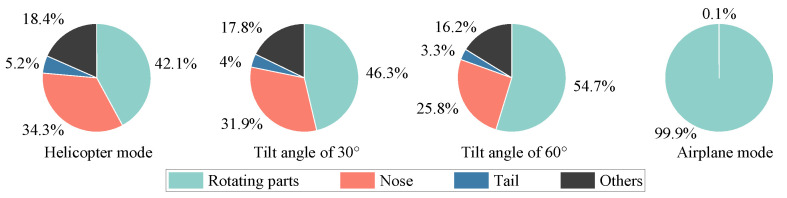
The contribution ratio of each scattering source to the total scattering, $\theta =\varphi =0^{\circ }$, f=10GHz.

Figure 6 shows the multi-mode dynamic RCS of tilt-rotor aircraft at an attitude of $\theta =\varphi =0^{\circ }$, and the wave frequency is 10 GHz. Figure 7 shows the contribution ratio of each scattering source to the total scattering. The tilt-rotor aircraft flies in helicopter mode from 0 to 0.15 s, in transition mode from 0.15 to 12.15 s, and in airplane mode from 12.15 to 12.3 s. The rotation period of the rotor is 0.15 s, and it takes 12 s for the tilt-rotor aircraft to change from helicopter mode to airplane mode. To make the RCS results clear in the images, the dynamic RCS images were drawn with a non-uniform sampling time interval of 0.000833 s in helicopter and airplane mode, and 0.0267 s in transition mode.

As can be seen in Figure 6, three distinct periodic features appear in the helicopter mode from 0 to 0.15 s. At the moments of 0.05, 0.1, and 0.15 s, the blade leading edges of the two rotors are perpendicular to the incident direction of the electromagnetic wave, at which time the RCS has a short-lived peak (leading edge peak) in the local range with a value of 17.36 dBsm. Since the angle between the blade’s leading edge and the trailing edge is 2°, the trailing edges are perpendicular to the wave at about 0.024, 0.074, and 1.024 s. And the RCS value at these moments is 17.09 dBsm, which is slightly lower than the leading edge peak. This is because the blade’s leading edge has a small curvature, and its echo characteristics are dominated by face reflection. While the trailing edge is a wedge configuration, the echo characteristics are mainly edge diffraction, which is a weaker scattering source compared to face reflection. In addition, the RCS shows a significant increasing and then decreasing trend in the intervals of [0.013, 0.024] s, [0.063, 0.074] s, and [0.113, 0.124] s, which is mainly caused by the gradually increasing and then decreasing multiple scattering between the lower surface of the forward blades and the upper surface of the nacelles. This multiple scattering leads to the maximum RCS in the helicopter mode, which is 20.81 dBsm. 

**Figure 8:**
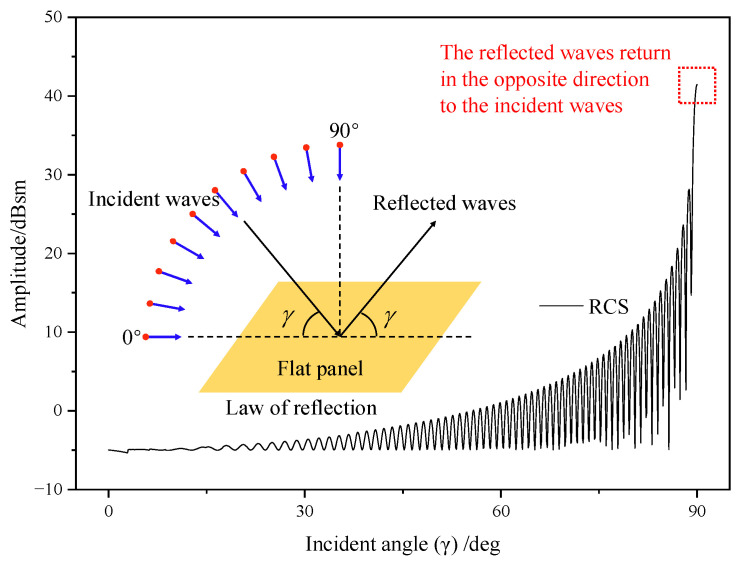
The law of reflection and the RCS characteristics of a flat panel.

In the transition mode, the leading edge peak should actually occur every 0.05 s, but due to the artificially changed sampling interval, the graph shows a leading edge peak every 0.4 s. As the transition proceeds, the RCS shows a sharp oscillation feature. And the leading edge peak is gradually obvious, whose value in the interval of [9.23, 12.15] s is much higher than the other sampling points in the local area. The rotors tilt from horizontal to vertical, while the upper surface of the rotors exposed to the radar wave changes from less to all, resulting in a rising RCS amplitude from 12.29 dBsm to 47.61 dBsm. It can be seen from Figure 7 that with the increase in tilt angle, the contribution of the rotating parts to the total scattering is also increasing. In airplane mode, the total scattering is almost entirely from the rotating parts. In non-airplane mode, the nose contributes more than a quarter of total scattering, and the other parts contribute about one-sixth; the tail contributes 3~5%. The physical mechanisms of the strong specular reflection on the rotor’s upper surface during the transition can be explained using the RCS characteristics of the flat panel. Figure 8 shows the RCS results of a flat panel scanned by a monostatic radar. The RCS of a flat panel gradually increases as the incident wave angle changes from 0° to 90°. This is because, on the panel surface, the incident wave angle (γ) is equal to the reflection angle (law of reflection). When the radar waves come from an angle of 90°, all the reflected waves return in the opposite direction from the incident waves. And at this time, the echo energy received by the receiver radar is the strongest, and the RCS is also the largest. The tilt-rotor can be compared to a flat panel; with the tilting of the rotor, more and more incident waves can return directly along the original path, so the RCS gradually increases. When the rotor tilts to 90°, the rotor disk is completely perpendicular to the incidence direction. Therefore, at this time, the specular reflection on the rotor’s upper surface is the strongest and the RCS is the largest. In the dashed black box of Figure 6, the RCS first rises and then falls, leading to a special region where the maximum RCS is 24.27 dBsm at 1.85 s (at this moment the tilting angle is 13°). The reason for this phenomenon is that the angle between the side line and axis of the conical hub is 13° in the present aircraft model, and at a tilting angle of 13°, the hubs are perpendicular to the incident wave and become strong scattering sources. Then, as the tilting angle deviates from 13°, the scattering intensity at the hubs decreases, as does the RCS amplitude.

In airplane mode, the upper surface of the rotors is completely perpendicular to the incident wave, creating an extremely strong specular reflection with a mean RCS of 47.71 dBsm. The dynamic RCS only fluctuates periodically in the range of [−0.36, +0.33] dB around the mean value, which is caused by the periodic shading of the rotors on the wings.

**Figure 9:**
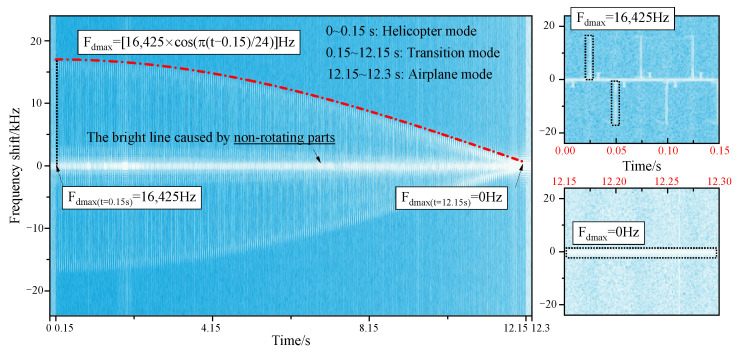
Multi-mode micro-Doppler of tilt-rotor aircraft, $\theta =\varphi =0^{\circ }$, f=10GHz.

**Figure 10:**
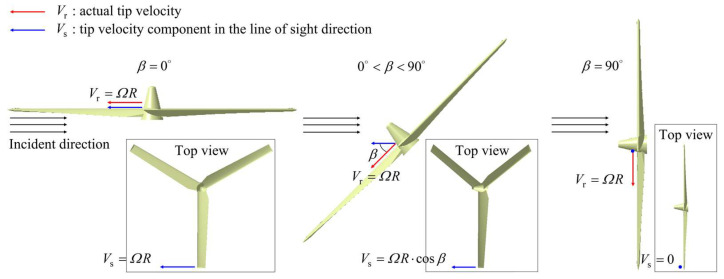
The influence of the rotors tilting on micro-Doppler effect.

Figure 9 reveals the micro-Doppler effects of tilt-rotor aircraft from helicopter mode to transition mode to airplane mode. When the aircraft has a translational velocity in the direction of the radar line of sight, the micro-Doppler will have an overall translation on the frequency shift axis, which is not considered in this paper. A positive micro-Doppler frequency shift is generated when the forward blades are perpendicular to the incident wave, and a negative shift is generated when the retreating blades are perpendicular to the incident wave. In helicopter mode, the maximum micro-Doppler frequency shift is kept at 16,425 Hz, and the scintillation period is 0.05 s. There is always a bright scintillation band distributed around the zero frequency, which is caused by the non-rotational parts of the tilt-rotor aircraft. In the transition mode, at the moment of 0.15 s, the maximum micro-Doppler frequency shift is 16,425 Hz, which is the same as that of helicopter mode. After the rotors start to tilt, the maximum micro-Doppler frequency shift decreases according to the law of

(18) $$F_{\mathrm{dmax}}=\left[16,425\cdot \cos\left(\frac{\pi \left(t-0.15\right)}{24}\right)\right]\left(\mathrm{Hz}\right),$$

until it drops to 0 Hz at the moment of 12.15 s. But the time interval between the two adjacent scintillation bands stays at 0.05 s. Figure 10 reveals the influence mechanism of the rotors tilting on the micro-Doppler effect. When the component of the blade linear velocity in the radar line of sight direction changes, the maximum micro-Doppler frequency shift also changes in response. In airplane mode, the micro-Doppler spectrum distribution is characterized by a bright line concentrated near the zero frequency because there is no velocity component in the direction of the radar line of sight. And it is not negligible that, if the forward speed of the tilt-rotor aircraft is taken into account, there will be a micro-Doppler frequency shift.

**Figure 11:**
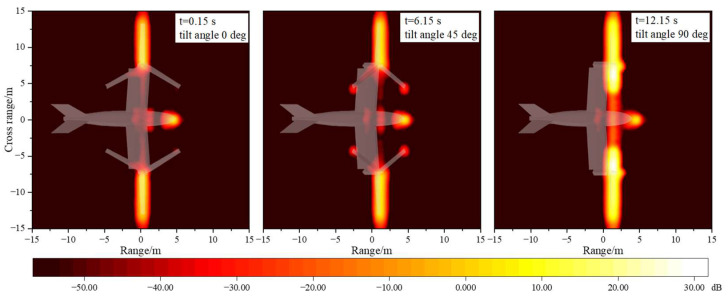
Multi-mode IASR images of tilt-rotor aircraft, $\theta =\varphi =0^{\circ }$, f=10GHz.

Figure 11 provides the multi-mode ISAR images of tilt-rotor aircraft. It can be seen that at the attitude of $\theta =\varphi =0^{\circ }$, the main strong scattering sources of the tilt-rotor aircraft are concentrated in the head, the wings, and the rotor blades, which are perpendicular to the incident wave. In the transition mode, the scattering intensity at the blade tips increases compared to the helicopter mode. When the tilting angle is 90°, the scattering intensity at the upper surface of the rotors increases rapidly and substantially. The results of these ISAR images are all consistent with the trends of the dynamic RCS profiles. And in order to improve the survivability of the tilt-rotor aircraft, it is necessary to focus on the stealth design of the highlighted areas in the ISAR images. However, it should be noted that when the tilt-rotor aircraft flies in airplane mode, it is very difficult to achieve the rotor’s stealth against the radar directly in front of the aircraft. At this time, stealth measures such as morphing rotor structures or radar-absorbing materials can be further considered.

### 3.2. Effects of Pitch Angle

**Figure 12:**
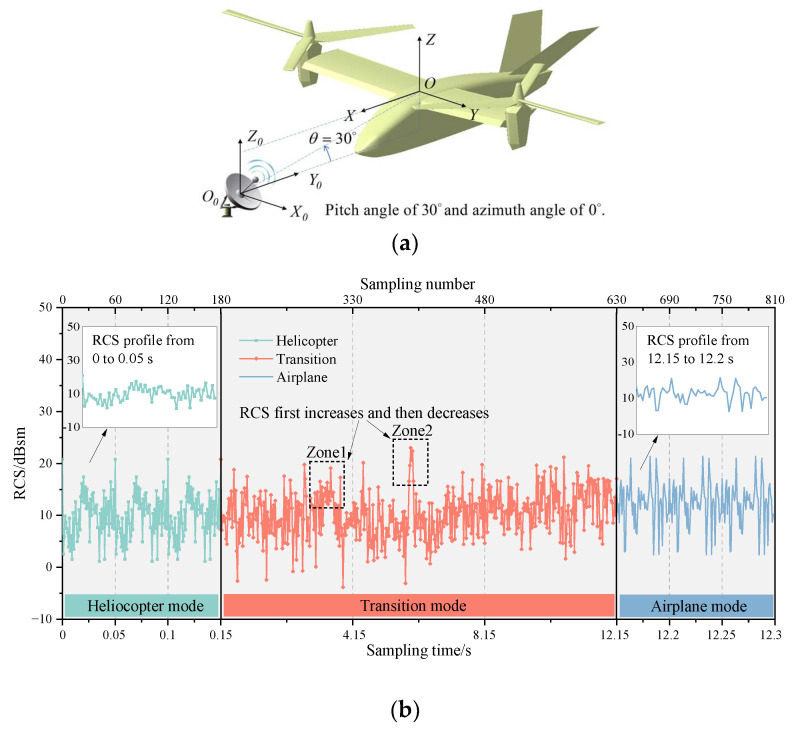
Multi-mode dynamic RCS of tilt-rotor aircraft, $\theta =30^{\circ }$, $\varphi =0^{\circ }$, f=10GHz. (**a**) Relative directional relationship between aircraft and radar; (**b**) dynamic RCS.

**Figure 13:**
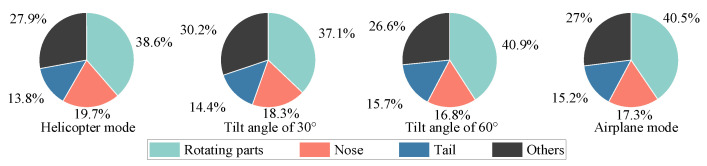
The contribution ratio of each scattering source to the total scattering, $\theta =30^{\circ }$, $\varphi =0^{\circ }$, f=10GHz.

In fact, tilt-rotor aircraft usually have a certain pitch or azimuth angle relative to the detection radar. Therefore, the effects of pitch angle and azimuth angle on the dynamic electromagnetic scattering of the tilt-rotor aircraft are investigated next. Figure 12 shows the multi-mode dynamic RCS of tilt-rotor aircraft at the attitude of $\theta =30^{\circ }$ and $\varphi =0^{\circ }$. Figure 13 shows the contribution ratio of each scattering source to the total scattering.

As can be seen in Figure 12, at a pitch angle of 30°, the dynamic RCS still possesses a significant periodic characteristic in the helicopter mode, and there are three cycles in 0.15 s. Due to the larger area of the lower surface of the rotors exposed to the radar incidence, the blade leading edge peak increases to a value of 20.81 dBsm. However, when the blade’s trailing edges are perpendicular to the incident wave, the RCS is reduced by 5.74 dB compared to the pitch angle of 0°. In addition, in the intervals of [0.013, 0.024] s, [0.063, 0.074] s, and [0.113, 0.124] s, the multiple scattering between the lower surface of the forward blades and the upper surface of the nacelles still becomes stronger first and then weaker, so the RCS increases first and then decreases.

In transition mode, the blade leading edge peak is no longer obvious, and the RCS increase is only 2.32 dB after the conversion from helicopter mode to airplane mode. And in the dynamic RCS profiles, there are two special zones where the RCS first increases and then decreases. “Zone1” is caused by the nacelles, and “Zone2” is caused by the hubs. These two components are both first gradually perpendicular to the direction of the incidence and then away from the direction during the tilting process, thus causing the two special zones.

In airplane mode, the RCS oscillation is much larger than that of the pitch angle of 0°, which is in the range of [−11.72, +7.25] dB around the mean value of 14.11 dBsm. The periodic features of the rotors’ scattering still exist in this mode, but the rotors are no longer the dominant scattering sources.

From the perspective of the contribution ratio of each scattering source to the total scattering, at different tilt angles, the contribution ratio of each source remains basically unchanged, and the contribution of the rotating parts is the largest. The tail contribution has been significantly improved compared to the state of 0° azimuth and pitch angle, accounting for about one-seventh.

**Figure 14:**
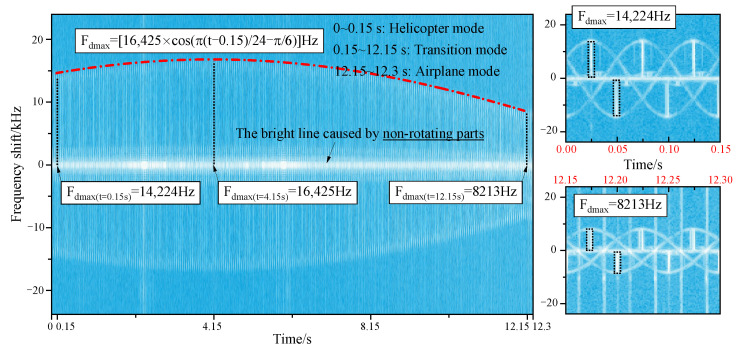
Multi-mode micro-Doppler of tilt-rotor aircraft, $\theta =30^{\circ }$, $\varphi =0^{\circ }$, f=10GHz.

Figure 14 reveals the multi-mode micro-Doppler of tilt-rotor aircraft at the attitude of $\theta =30^{\circ }$ and $\varphi =0^{\circ }$. It can be seen that the scintillation period remains unchanged in all modes, which is always 0.05 s. But the patterns of the maximum micro-Doppler frequency shift have changed significantly. Since the pitch angle is 30°, the angle between the incidence and the rotor disk is 30° in helicopter mode, and the maximum micro-Doppler frequency shift of helicopter mode needs to be multiplied by $\cos30^{\circ }$, which is 14,224 Hz. In transition mode, the angle between the incidence and the rotor disk is equal to the tilting angle minus the pitch angle. So, after the rotors start to tilt, the maximum micro-Doppler frequency shift is

(19) $$F_{\mathrm{dmax}}=\left[16,425\cdot \cos\left(\frac{\pi \left(t-0.15\right)}{24}-\frac{\pi }{6}\right)\right]\left(\mathrm{Hz}\right).$$

At the moment of 4.15 s, we have $F_{\mathrm{dmax}}=16,425\mathrm{Hz}$. From 12.15 s to 12.3 s, the angle between the incidence and the rotor disk is kept at 60°; hence, the maximum micro-Doppler frequency shift of the airplane mode is 8213 Hz.

**Figure 15:**
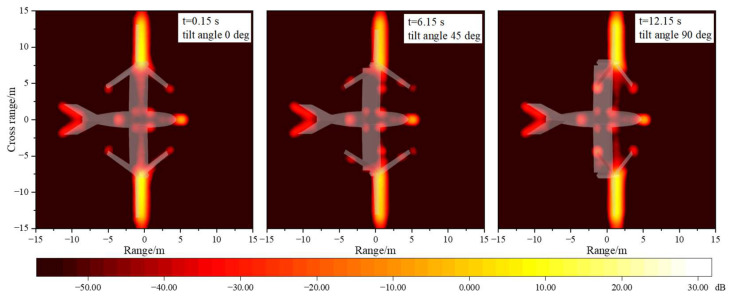
Multi-mode ISAR images of tilt-rotor aircraft, $\theta =30^{\circ }$, $\varphi =0^{\circ }$, f=10GHz.

Figure 15 provides the multi-mode ISAR images of tilt-rotor aircraft at the attitude of $\theta =30^{\circ }$ and $\varphi =0^{\circ }$. It can be seen that when the pitch angle is 30°, the surface areas of the tilt-rotor aircraft exposed to the electromagnetic rays are larger, resulting in an increase in the scattering intensity at the belly, the tail wings, and the wing–fuselage junctions. Because of the V-shaped construction, the contribution of the tail wings to the total scattering is dominated by edge diffraction. The wing–fuselage junctions produce multiple scattering due to the presence of angular structures, which should be avoided as much as possible in the stealth design of tilt-rotor aircraft. Furthermore, at this pitch angle, the rays do not have the opportunity to irradiate vertically on the rotor disk, so the scattering intensity at the blade’s surface does not differ much in different modes.

### 3.3. Effects of Azimuth Angle

**Figure 16:**
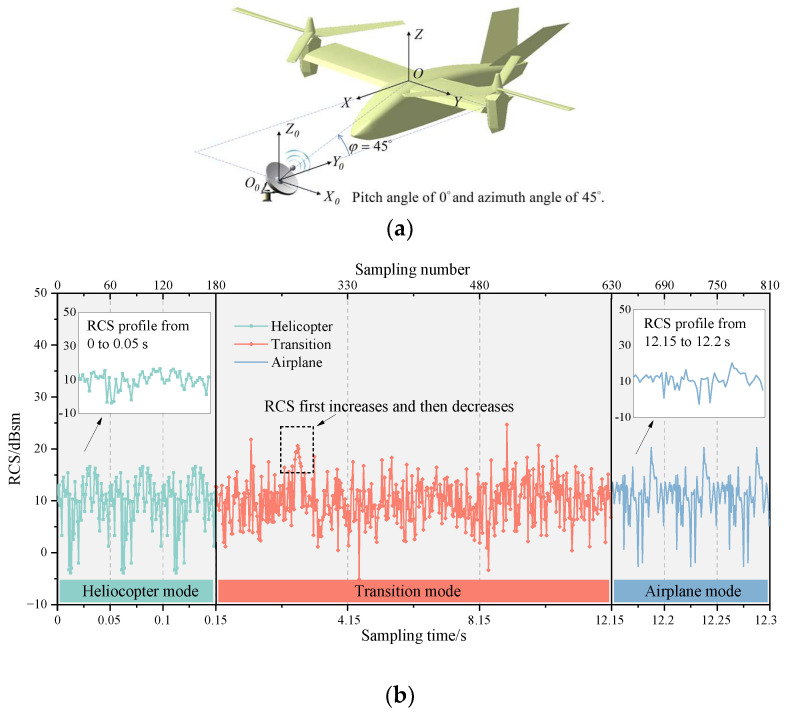
Multi-mode dynamic RCS of tilt-rotor aircraft, $\theta =0^{\circ }$, $\varphi =45^{\circ }$, f=10GHz. (**a**) Relative directional relationship between aircraft and radar; (**b**) dynamic RCS.

**Figure 17:**
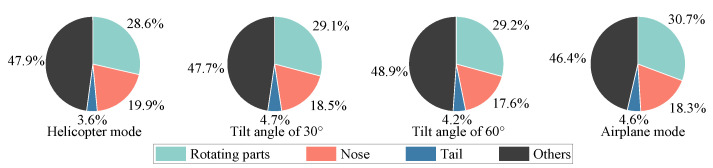
The contribution ratio of each scattering source to the total scattering, $\theta =0^{\circ }$, $\varphi =45^{\circ }$, f=10GHz.

Figure 16 shows the multi-mode dynamic RCS of tilt-rotor aircraft at the attitude of $\theta =0^{\circ }$ and $\varphi =45^{\circ }$. Figure 17 shows the contribution ratio of each scattering source to the total scattering.

Due to the azimuth angle of 45°, the rotational phase of the two rotors in the direction of the radar line of sight is no longer symmetrical. As shown in Figure 16, in the helicopter mode, there are six cases in which the blade leading edge is perpendicular to the incident wave. These cases are distributed at the moments of 0.01875 s, 0.03125 s, 0.06875 s, 0.08125 s, 0.11875 s, and 0.13125 s, and the RCS values alternate at 7.99 dBsm and 13.53 dBsm. These two values are less than the blade leading edge peak at the state of the 0° azimuth angle because the leading edges of the two rotors are no longer perpendicular to the direction of the incident wave at the same time, resulting in a reduction in the total scattering intensity. Therefore, when the rotational phase is not symmetrical relative to the incident wave, the influence of the periodic rotation of the rotors on the total scattering is weakened. 

In transition mode, the dynamic RCS still fluctuates dramatically, but it is difficult to directly find out the effects of the rotors’ periodic rotation only from the RCS profiles. And the RCS increase is only 0.92 dB after the conversion from helicopter mode to airplane mode. During the tilting process, the hubs are perpendicular to the incident wave at about 2.63 s, which will produce a peak value in the local area, so the RCS increases first and then decreases near this moment.

In airplane mode, the RCS oscillation is in the range of [−15.15, +7.79] dB around the mean value of 12.49 dBsm. And except for the numerical differences, the overall features of the dynamic RCS are similar to those at the state of 30° pitch angle.

In addition, due to the change in azimuth angle, the incident wave cannot be vertically incident on the rotor disk plane, so the contribution of the rotating parts to the total scattering is greatly reduced. The multiple scattering between the various components is enhanced, and the scattering intensity of the tail is still maintained at a very low level.

**Figure 18:**
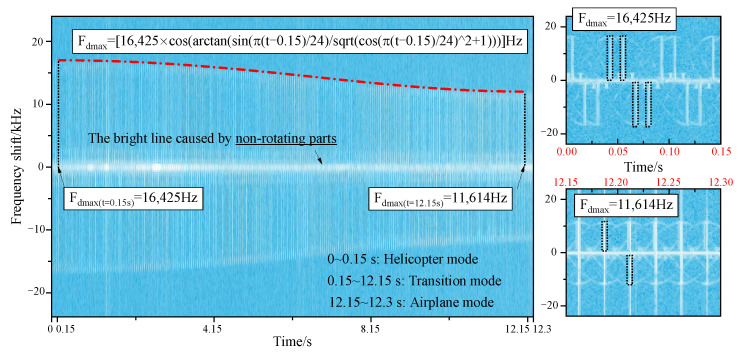
Multi-mode micro-Doppler of tilt-rotor aircraft, $\theta =0^{\circ }$, $\varphi =45^{\circ }$, f=10GHz.

Figure 18 reveals the multi-mode micro-Doppler of tilt-rotor aircraft at the attitude of $\theta =0^{\circ }$ and $\varphi =45^{\circ }$. It can be seen that with the change of azimuth angle, there is a time difference of 0.0125 s between the two rotors’ micro-Doppler scintillation characteristics. However, due to the constant rotation period of the rotors, the scintillation period is always maintained at 0.05 s. In the helicopter mode, the maximum micro-Doppler frequency shift is the same as that of the state of $\theta =\varphi =0^{\circ }$, which is 16,425 Hz. This is because only the azimuth angle is changed, and in fact, the angle between the incident wave and the rotor disk is kept at 0°. In the transition mode, at the state of $\theta =0^{\circ }$ and $\varphi =45^{\circ }$, the angle between the incidence wave and the rotor disk can be expressed as $\theta_{0}=\arctan\left(\frac{\sin\beta \left(t\right)}{\sqrt{{\left(\cos\beta \left(t\right)\right)}^{2}+1}}\right)$, where $\beta \left(t\right)$ is the tilting angle. So, the maximum micro-Doppler frequency shift is

(20) $$F_{\mathrm{dmax}}=\left[16,425\cdot \cos\left(\arctan\left(\frac{\sin\frac{\pi \left(t-0.15\right)}{24}}{\sqrt{{\left(\cos\frac{\pi \left(t-0.15\right)}{24}\right)}^{2}+1}}\right)\right)\right]\left(\mathrm{Hz}\right).$$

When the rotors tilting is finished, the $\theta_{0}$ is kept at 45°; hence, the maximum micro-Doppler frequency shift of the airplane mode from 12.15 s to 12.3 s is 11,614 Hz. Moreover, due to the particularity of the 45° azimuth angle, both positive and negative frequency shifts appear on the micro-Doppler spectrum, which means that there are forward and retreating blades perpendicular to the direction of the incident wave at the same time.

It can be seen from the above analyses that the tilt-rotor aircraft has rotors that can be converted from a horizontal state to a vertical state, so its micro-Doppler shift can produce a large and continuous variation between zero shift and maximum shift, which is a feature not found in other types of vehicles. By observing the range of micro-Doppler shift variation, it is possible to identify whether the vehicle is a tilt-rotor aircraft. In addition, the tilt-rotor aircraft can fly at any tilt angle of the rotor. And in order to maintain a stable flight attitude, there is a certain relationship between the tilt angle of the rotor and the flight speed. That is, at a certain tilt angle of the rotor, the tilt-rotor aircraft can only safely fly at a certain range of speed. Therefore, according to the measured micro-Doppler shift, the tilt angle of the rotor can be calculated through the shift variation functions. And furthermore, the flight mode can be confirmed and the flight speed estimated.

**Figure 19:**
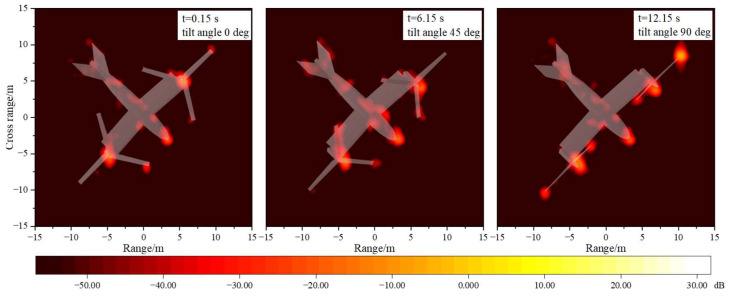
Multi-mode ISAR images of tilt-rotor aircraft, $\theta =0^{\circ }$, $\varphi =45^{\circ }$, f=10GHz.

Figure 19 gives the multi-mode ISAR images of tilt-rotor aircraft. The strong scattering sources of the tilt-rotor aircraft at the state of $\theta =0^{\circ }$ and $\varphi =45^{\circ }$ are mainly distributed in a point-like manner, concentrated in the parts of the nose, the hubs, the blade tips, the tail wings, and the fuselage side. The scattering intensity of each part at different tilt angles is relatively consistent, and there is no case where the scattering intensity of one part is much larger than that of other parts. It also shows that the tilt-rotor aircraft can maintain a more stable electromagnetic scattering level when the radar wave is incident from an oblique direction in the horizontal plane.

## 4. Conclusions

In this paper, a dynamic simulation method for the scattering characteristics of the tilt-rotor aircraft is established by coupling SBR, UTD, STFT, IASR imaging, and a time-varying mesh approach. By studying the multi-mode RCS, micro-Doppler, and microwave images of tilt-rotor aircraft, the following conclusions could be drawn:

(1) Due to the rotation of the rotors, the dynamic RCS of the tilt-rotor aircraft in helicopter mode and airplane mode has obvious periodic characteristics. When the radar wave is incident from the front of the aircraft, the conversion from helicopter mode to airplane mode makes the rotor disk gradually perpendicular to the incident wave, resulting in an extremely strong specular reflection and a large increase in RCS, with an increase of 35.32 dB. In order to cope with the case, the rotor’s upper surface is suggested to be coated with wave-absorbing material to weaken the strong specular reflection. When the pitch angle or azimuth angle is not zero, the effect of the transition mode on the RCS growth is rapidly weakened.
(2) When the forward or retreating blades are perpendicular to the incident direction of radar rays, the tilt-rotor aircraft has the maximum micro-Doppler frequency shift. In helicopter mode and airplane mode, the maximum micro-Doppler frequency shift remains constant. As the tilting proceeds, the angle between the rotor disk and the radar wave changes, which is related to the flight time, the tilt speed, and the wave’s incident direction. This angle has a definite function expression, and the maximum micro-Doppler frequency shift is proportional to the cosine of this angle. By acquiring real-time micro-Doppler signals from an airborne target and analyzing their changing patterns, it can be determined whether the target is a tilt-rotor aircraft. If so, the aircraft’s flight mode and speed are able to be rapidly identified according to the shift amplitude.
(3) The tilt-rotor aircraft has multiple important scattering sources. The surfaces of the rotors, the leading edge of the wings, and the nose show a planar scattering source distribution. While the blade tips, the wing–fuselage junctions, and the tail wings show a point-like distribution. In different flight modes and attitude angles, the importance of the above scattering sources is different. When the radar wave is incident from the front of the tilt-rotor aircraft, which flies in airplane mode, the upper surface of the rotor disk is the most significant strong scattering source, and the RCS reduction is needed in this area. Therefore, according to the flight mission requirements of the tilt-rotor aircraft, a reasonable analysis of the key scattering areas can provide effective help for the stealth design of the whole aircraft (e.g., blade tip sharpening, wing–fuselage integration fusion design, tail parallel swept design, and so on).

## Figures and Tables

**Table 1:** Main parameters of the tilt-rotor aircraft model.

Parameters	Values	Parameters	Values
Radius of rotors (R)	5.9 m	Length of fuselage (L)	17.25 m
Rotational speed (Ω)	400 r/min	Width of fuselage ($W_{F}$)	2.17 m
Wingspan (W)	7.4 m	Height of tail wings (H)	4.20 m

## Data Availability

No new data were created.
